# Age-Specific Mechanisms in an SSVEP-Based BCI Scenario: Evidences from Spontaneous Rhythms and Neuronal Oscillators

**DOI:** 10.1155/2012/967305

**Published:** 2012-12-06

**Authors:** Jan Ehlers, Diana Valbuena, Anja Stiller, Axel Gräser

**Affiliations:** ^1^Institute of Automation (IAT), University of Bremen, P.O. Box 278, 28359 Bremen, Germany; ^2^Institute of Psychology and Cognition Research (IPK), University of Bremen, P.O. Box 330440, 28359 Bremen, Germany

## Abstract

Utilizing changes in steady-state visual evoked potentials (SSVEPs) is an established approach to operate a brain-computer interface (BCI). The present study elucidates to what extent development-specific changes in the background EEG influence the ability to proper handle a stimulus-driven BCI. Therefore we investigated the effects of a wide range of photic driving on children between six and ten years in comparison to an adult control group. The results show differences in the driving profiles apparently in close communication with the specific type of intermittent stimulation. The factor age gains influence with decreasing stimulation frequency, whereby the superior performance of the adults seems to be determined to a great extent by elaborated driving responses at 10 and 11 Hz, matching the dominant resonance frequency of the respective background EEG. This functional interplay was only partially obtained in higher frequency ranges and absent in the induced driving between 30 and 40 Hz, indicating distinctions in the operating principles and developmental changes of the underlying neuronal oscillators.

## 1. Introduction

Bioelectrical oscillations recorded with the electroencephalogram (EEG) can be classified with regard to the relation of stimulation [1]. Accordingly, oscillatory activity can either be unrelated to any kind of external release (spontaneous background activity) or, as opposed to this, time-locked to situational events. Intermittent photic stimulation (IPS) of variable frequency at a rate of 4 Hz or higher evokes a synchronized cortical response consisting of rhythmic activity linked to the stimulus at a frequency identical or harmonically related to that of the triggering event [2, 3]. EEG activity that arises from such repetitive stimulation is known as steady-state visual evoked potentials (SSVEPs). It appears maximal over posterior regions of the head and can be identified as sharp peaks in the amplitude-frequency spectrogram of the EEG [4]. The general acceptance of a resonance nature of induced driving responses is well documented and was experimentally confirmed by several investigators [5]. Most likely, the described effects occur due to neural oscillators which preferably oscillate at specific frequencies, so-called resonance frequencies [3]. Though it is accepted that IPS enhances the resonance properties of EEG oscillators, the underlying mechanisms of induced driving responses continue to be debated. In all probabilities the resonance emerges from different neural circuits that bear various functional roles whereby it is assumed that SSVEP activity rather arises from stimulus-induced phase resetting within the dynamics of the ongoing EEG than from additive amplitude modulation [6–9].

Experience has shown that the resonance phenomenon evolves extremely selective with stronger responds to predetermined frequencies. A gradual increase of stimulation frequencies across the entire EEG range allows to calculate an individual characteristic of reactivity and a response profile based on the peaks elicited in the EEG spectrogram [2]. Herrmann [3] reports on pronounced cortical reactions to flickering stimuli in the 10, 20, 40, and 80 Hz range compared to adjacent frequencies. According to [10] the amplitude of the SSVEP in occipital regions peaks at 15 Hz. Though these oscillators appear to be stable over a long period [11], former investigations observe a large interindividual variability of the driving response [5], a finding that may contribute to the varying results in smaller samples. Nevertheless, a large number of studies (among others [2, 11, 12]) depict that the EEG photic driving is positively correlated with the spontaneous alpha power spectral, meaning a maximum increase in amplitude during stimulation near the dominant resting EEG frequency (DRF). Though the precise mechanism of action constituting the functional interaction between driving profile and ongoing EEG activity is not yet understood, the immediate proximity of preferred resonance frequencies to the peak alpha activity suggests the assumption that both rhythms share similar operating principles in synchronizing neural activity [12]. Birca et al. [13] do not find a considerable correlation between the dominant frequency in the resting EEG and the frequency of the IPS which elicited the best driving response. However, similar to other studies [3, 14] they report that two out of three subjects that feature a resting alpha peak surrounding 10 Hz revealed a preferred resonance frequency at this very wave. Moreover, most investigators consistently report that IPS near the peak alpha frequencies of the background EEG suppresses the spontaneous EEG activity in terms of a sharp decrease in amplitude at exactly the frequency of the individual background alpha peak [2, 15].

Given that the photic driving interacts with the composition of the ongoing EEG as illustrated above, the functional interplay should be influenced by development-specific changes within the background EEG. It is known that the interindividual variability of quantitative EEG parameters increases with age [16] and there are marked changes in the (relative) band power during cognitive development. Thereby it is generally believed that brain maturation is associated with a substitution of slow activity by a faster, particularly, decrease in the lower frequency range (delta and theta) and a continuous—though not equable—increase in faster bands (alpha and beta), since the development of the EEG is usually nonlinear [17]. Matthis et al. [18] observe the closest correlation with age in the relative amount of activity in the fast alpha band. This is accompanied by the finding that the individual occipital alpha rhythm frequency increases from around 8 Hz in 3–5-year-old children to approximately 10 Hz in subjects older than 10 years of age. Still, quantifying the magnitudes of elicited SSVEP responses led to no considerable age-related changes in children older than 3 years of age, whereas phase alignment values showed a gradual increase with age over occipital regions [13].

IPS is one of the most important functional tests used in the clinical EEG examination especially for detecting photoparoxysmal responses in the epileptic population [19]. Beyond, utilizing changes in SSVEPs is an established approach to operate a brain-computer interface (BCI). In the course of this a subject shifts his/her visual attention to sources of light that oscillate at different constant frequencies, respectively. As depicted above, focusing a flickering stimulus exhibits frequency-specific photic driving that can be detected over occipital areas and subsequently translated into a specific command [20]. This specific visual attention-based BCI approach has been successfully validated in different series of tests on healthy subjects [20–24] and is currently adapted to disabled users in the EU-project BRAIN (http://www.brain-project.org/) (for first beginnings see [25, 26]). The latter efforts correspond to the classic goal of BCI research and make up the principal focus of most research groups, to provide severely disabled users with communication and control [20]. However, a systematic investigation of the achievements of varying young age groups in an SSVEP-based BCI scenario against the background of the described physiological mechanisms has—to our knowledge—not been conducted yet.

The principal goal of the present study was to assess to what extent development-specific changes in the background EEG influence the ability to proper handling of a stimulus-driven BCI software at an early stage. This implies that we are giving priority to both, the individual BCI performance in terms of the grade of accuracy as well as a possible causal connection to age-dependent dynamics in the oscillatory activity with emphasis on the associated varying frequency synchronization. To get to the bottom of these coherences we are investigating the effects of a wide range of photic driving, divided into three blocks of stimulation: a low frequency part that covers the common alpha range (7–11 Hz), a medium frequency section (13–17 Hz) that is known to produce prominent SSVEP responses and therefore is mostly consulted in corresponding publications [3, 10, 20, 27] and a high frequency range (30–48 Hz) that is investigated as of recently since such a fast repetitive stimulation above 30 Hz is less visual annoying and diminishes user fatigue as well as the risk of epileptic seizures for photosensitive subjects [28, 29]. As a processing algorithm we consulted the Bremen BCI, a software module that was tried and tested during several series of tests in the past [20, 24, 30]. The user application consisted of a monitor-based spelling device that was evaluated in previous studies and enables the user to write texts by successive selecting single characters [24, 30].

## 2. Methods and Materials

### 2.1. Subjects

A total of 51 adults and children aged between six and 33 years were included in the present cross-sectional study.  Table 1 depicts (among physiological properties) the male/female ratios and the distribution of participants in the various age groups. All subjects were right handed and had normal or corrected-to-normal vision and no prior experience with BCIs. Inclusion in the study was based on the following criteria: no obvious somatic disease, no history of head injury, no neurological or psychiatric disorder, and no drug-related illness. No participant was taking any form of medication at the time of testing. All subjects were informed that the repetitive visual stimulation might lead to epileptic seizures and confirmed that they had never suffered from epilepsy or various photosensitive reactions. The study was approved by the Ethics Committee of the University of Bremen. A written informed consent was obtained from the adult participants (psychology students) and the legal guardians of the children.

### 2.2. Display and Procedure

**Figure 1:**
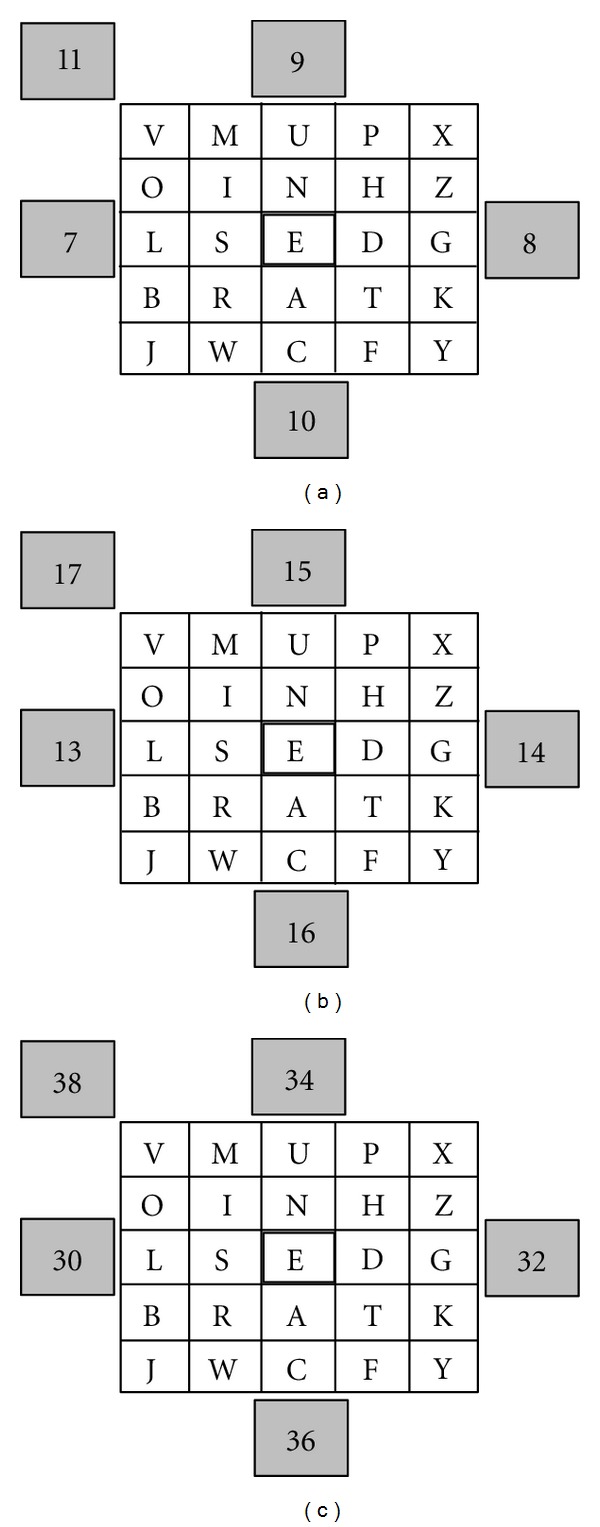
Display with LEDs surrounding the letter field of the spelling device. Figures refer to the respective flickering frequency in Hz. (a) Low frequency stimulation. (b) Medium frequency stimulation. (c) High frequency stimulation. The cursor is positioned over the E.

The adult subjects came to the laboratory during the morning hours whereas the children were tested at preferably corresponding times in a work room of their basic school, both environments exhibiting a high level of background noise. To ensure a comparable contrast of the flickering stimuli, the lightning conditions were kept similar in both surroundings: true light but no direct sunlight. Subjects were seated in comfortable chairs about three ft. from a 17′′ BENQ Q7T5 LCD monitor with a 60 Hz refresh rate. A quadratic frame equipped with five rectangular light-emitting diodes (LEDs) (four positioned in the middle of each edge, the fifth at the upper left corner) was mounted to the screen, consequently surrounding the displayed letter field. Each LED had an edge length of 20 × 14 mm. The specific oscillations were controlled by a microcontroller (PIC16F877, Microchip, Chandler, Arizona, USA). The speller arrangement was determined through prior work [30, 31]: the characters were arranged regarding their incidence in the German alphabet with rarely used letters at the periphery and E (commonest) at the exact center. At the beginning of each run, the cursor was presented at this very position.  Figure 1 depicts the consulted display. Each LED was associated with a specific command, in particular left, right, up, down and select (the top left LED). Depending on the testing condition the five LEDs oscillated with assigned frequencies: 7, 8, 9, 10, and 11 Hz (in the following referred to as “low frequency stimulation”), 13, 14, 15, 16, and 17 Hz, (in the following referred to as “medium frequency stimulation”) and 30, 32, 34, 36, and 38 Hz (in the following referred to as “high frequency stimulation”). Subjects were instructed to spell by focusing exactly the LED that is associated with the desired command, meaning to move the cursor up, down, left, or right. A character highlighted by the cursor could be selected by focusing the LED coupled with the select*‌* command. The Bremen BCI software automatically determines the best spatial filter for each subject and subsequently computes the signal-to-noise ratio (SNR) for each of the various stimulation frequencies [30]. If the SNR at a specific frequency exceeds a defined threshold, the corresponding command is executed (for details on the processing algorithm see Section 2.4). To clarify this by an example in the medium frequency condition (Figure 1(b)) and to navigate the cursor upwards, subjects had to focus the upper LED. If the corresponding frequency activity (15 Hz) exceeds the predetermined threshold, the command is executed; if, for example, correspondingly high values are detected for 17 Hz, the currently highlighted character is selected. The bottom edge of the screen displayed the letter string that was already picked. Except for the group of the youngest subjects (sample 1: *⌀* 6.73 yrs) all participants used the present BCI system to spell six words, respectively, two in each of the three conditions (low, medium, and high frequency induced driving). The terms of stimulation were determined randomly throughout the testing. The word material was selected considering two premises: an age appropriate composition and a uniform distribution of the involved commands; as a result it was guaranteed that all frequencies contribute just about equally to the spelling. An entire session (including the preparation stage) took about 45 minutes. During a practice phase prior to the study as such, we provided the opportunity to spell the subjects name to ensure that the operating principle of the SSVEP-based system was figured out. Every experimental run ended just as the subject spelled the desired phrase (regardless of whether accurate or approximately correct) or chose to stop spelling. However, no run took less than two minutes and at least 20 commands per word were executed. The youngest subjects (sample 1: *⌀* 6.73 yrs) followed an identical operating procedure, however; due to age-related spelling proficiency they were assigned to different word material and spelled only one term per condition. To ensure that eventual group differences within the performance exclusively result from the endogenous frequency development instead from age-specific deficits in visual searching abilities, the younger subjects were assisted by the investigator regarding character or LED selection. Prior to the SSVEP investigation we recorded the resting EEG during relaxed wakefulness with the subjects focusing a fixation cross for one minute and afterwards keeping their eyes closed for the same limited period.

### 2.3. Data Collection

EEG data was recorded from the surface of the scalp via eight sintered Ag/Ag-Cl EEG electrodes. AF_Z_ served as ground; the input electrodes P_Z_, PO_3_, PO_4_, O_1_, O_Z_, O_2_, O_9_, and O_10_ were mounted according to the international 10–20 system of electrode placement [32]. Standard abrasive electrolytic electrode gel was applied; shielded cables connected the electrodes and the high impedance amplifier system (Porti32, Twente Medical Systems International). The sampling frequency was 2048 Hz; during the EEG acquisition a high-pass filter at 0.1 Hz was applied and a digital FIR low-pass filter at 552.96 Hz (0.27 × sampling rate) was directly applied in the amplifier. The general-purpose software platform BCI2000 [33] was consulted for data acquisition, storage, and real-time data processing. The SSVEP signal processing module (Bremen BCI software; see next chapter) was implemented in the BCI2000 framework.

### 2.4. Online Calculations: The Bremen BCI

The Bremen BCI signal processing algorithm is implemented in C++ and programmed for detecting SSVEP activity in a BCI scenario. Friman et al. [30] proposes multichannel signal detection for SSVEP applications using the following linear model that decomposes the measured signal *y*_*i*_(*t*) into three parts:

(1) $$\begin{array}{c} y_{i}\left(t\right)=\sum_{k=1}^{N_{h}}a_{i,k}\sin\left(2\pi kft+\phi_{i,k}\right)+\sum_{j}b_{i,j}z_{j}\left(t\right)+e_{i}\left(t\right). \end{array}$$

The first part of this model is the evoked SSVEP response signal modeled as a number of sinusoids with frequencies given by the stimulus frequency *f* and a number of harmonic frequencies *N*_*h*_, and the corresponding amplitude *a*_*i*,*k*_ and phase *ϕ*_*i*,*k*_. The second part describes the background brain activity and nuisance signals *z*_*j*_(*t*), which are added to each electrode signal and scaled with the weight factor *b*_*i*,*j*_. The nuisance signals are concurrent brain processes or external disturbances such as breathing artifacts and power line interference. The last part *e*_*i*_(*t*) describes a noise component in the measurement, which is specific for electrode number *i*.

In this work, eight input electrodes were used to record the neural activity from the occipital region of the scalp. To ensure a proper performance of analysis the recorded electrode signals are combined into channel signals [30]. For this, Bremen-BCI uses the minimum energy combination (MEC), a spatial filter that readjusts the input channels in order to minimize nuisance influence. As a result, electrodes with insufficient contact to say electrodes that transmit poor signals receive a low weighting or might even be ignored. Moreover, the combination matrix is constantly adapted to change the signal quality over time. This procedure is being executed every 125 ms. To provide sufficient EEG data for a proper analysis, classification is always based on a 2 s sliding window showing the recorded data in steps of 125 ms. In other words, the system stimulates the subject with a certain frequency and estimates the signal power that lies on each channel *s*_*l*_ and in the *k*th SSVEP harmonic frequency, as

(2) $$\begin{array}{c} {\hat{P}}_{k,l}={||X_{k}^{T}s_{l}||}^{2}, \end{array}$$

where *X* contains the sine and cosine pairs with the SSVEP harmonic frequencies. The test statistic, which is an average of the power over all *N*_*s*_ spatially filtered components and all *N*_*h*_ SSVEP harmonic frequencies, for testing the presence of an SSVEP response can be calculated by

(3) $$\begin{array}{c} T=\frac{1}{N_{s}N_{h}}\sum_{l=1}^{N_{s}}\sum_{k=1}^{N_{h}}{\hat{P}}_{k,l}. \end{array}$$

This procedure concludes in receiving one single value every 125 ms, implying that the system calculates one absolute value for every examined frequency. The last step of signal processing is the normalization for converting absolute values into relative values in order to yield comparability.

### 2.5. Statistical Analyses

A review of the empirical distribution of our data basis consulting the Kolmogorov-Smirnov test revealed that the behavioral data set (accuracy rates) is not well modeled by a normal distribution. Beyond, a Levene test revealed that the degree of variance homogeneity is not consistently adequate. Since the various sample sizes do not meet the requirements to still conduct a parametric test, we used separate the Kruskal-Wallis one-way analysis of variance followed by the Games-Howell post hoc tests to examine the effect of age on the induced driving responses within all three experimental conditions. To determine distinctions regarding the physiological data (DRF) we consulted a two-way analysis of variance (ANOVA) with the repeated-measures factor electrode position and a between-subjects factor age group. The Greenhouse-Geisser procedure for violations of the sphericity assumption was applied. Paired *t*-tests were conducted to isolate considerable differences; the significance levels were adjusted using the Bonferroni correction.

## 3. Results

### 3.1. Behavioral Data

**Figure 2:**
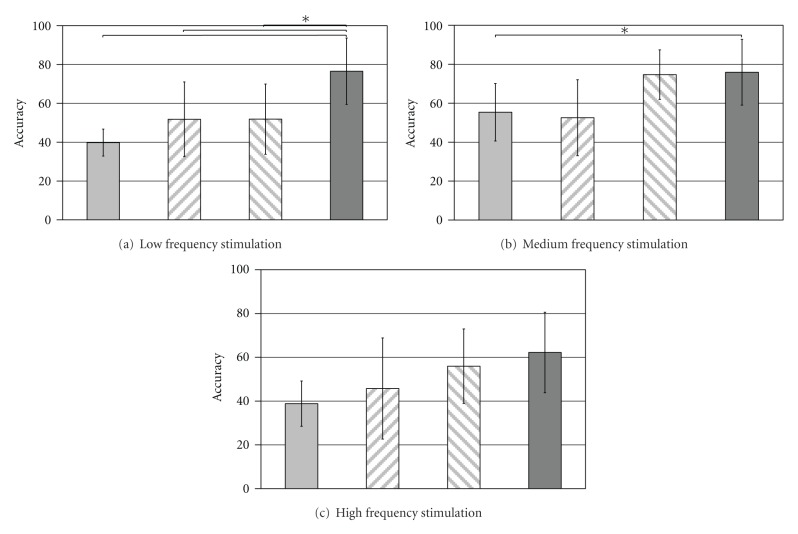
Mean accuracy rates during various frequency stimulations for all consulted age groups (light grey bar: group 1 (*⌀* 6.73 yrs); left hatched bar: group 2 (*⌀* 8.08 yrs); right hatched bar: group 3 (*⌀* 9.86 yrs); dark grey bar: group 4 (*⌀* 22.36 yrs)). The star depicts significant group differences.

Figure 2 depicts the mean accuracy rates of all consulted age groups during the varying kinds of stimulation. The parameter “accuracy” is defined as the correct-to-complete commands ratio within a single run. To ensure a comprehensive data record we also consulted aborted attempts (unfinished words) since the number of completed tasks was especially in the younger groups considerably small. More precisely, the total number of cancelled tasks (independent from the applied frequency set) steadily decreases with age: while the youngest participants (group 1: *⌀* 6.73 yrs) broke off 79% of all attempts (group 2: 71%; group 3: 41%), the adult subjects (group 4: 22.36 yrs) cancelled only 38% trials. Arranged according to frequency sets we ascertain the slightest group differences in the high frequency spectrum. In this experimental condition all age groups exhibit comparable high drop-out rates. In contrast, working with low frequencies leads to considerable effects in terms of continuously declining drop-outs rates with age (*χ* ^2^(3) = 9.011;  *P* < .05). However, the post hoc analysis specified no significant group differences.

Analyzing the individual accuracy rates yielded stable age effects, obviously determined by the respective set of frequencies (Figure 2). We observe a significant age group effect within the low frequency stimulation condition (Figure 2(a)) (*χ* ^2^(3) = 19.034;  *P* < .001). The adults obtain consistently higher accuracy rates compared to all three children samples (versus group 1: mean difference MD = 33.05; *P* < .001; versus group 2: MD = 27.39; *P* < .01; versus group 3: MD = 24.40; *P* < .01). In contrast, working with the medium frequency range based system leads to considerable differences only between the adults and the youngest sample (Figure 2(b)) (MD = 19.65; *P* < .05). Finally, we discover no significant age group distinctions on the basis of high frequency stimulation (Figure 2(c)). A comparison of the various stimulation frequencies within the “low” frequency range (7, 8, 9, 10, and 11 Hz) revealed a distinct effect for the adult sample: working on the basis of 10 or 11 Hz stimulation is accompanied by consistently higher accuracy rates compared to the results of 7 Hz (*χ* ^2^(4) = 22.454; *P* < .001) (versus 10 Hz: MD = 39.55; *P* < .001; versus 11 Hz: MD = 40,13; *P* < .001). Due to the smaller number of given commands within the younger groups, a similar subdivision of accuracy rates for every frequency is possible only to a limited extent. Nevertheless, on average all children samples also achieve higher accuracy rates on the basis of 10 and 11 Hz stimulation compared to their performance with 7, 8, or 9 Hz. However, there has as yet been no statistical confirmation.

### 3.2. Physiological Data

**Figure 3:**
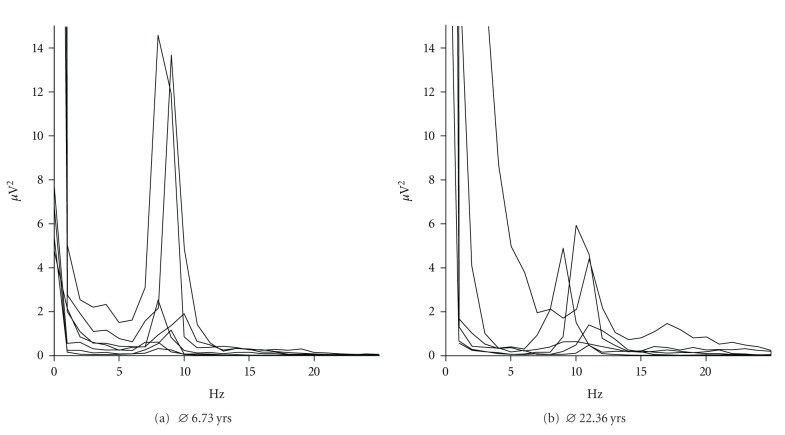
Spectrogram of eyes-closed occipital alpha synchronisation depicting representative subjects of age group 1 (a) and 4 (b).

The DRF peak in the alpha range could easily be determined in all test groups as exemplarily diagrammed in Figure 3 for group 1 (A) and 4 (B). The spectrograms depict the overlapping signal curves of representative subjects, indicating an age-specific shift in the peak synchronisation frequency.  Table 1 encloses a listing of mean values of the DRFs at selected occipital locations for all age-groups (except sample 2). It becomes apparent that the DRF increases from about 8-9 Hz (group 1: *⌀* 6.73 yrs) to 9-10 Hz (group 3: *⌀* 9.86 yrs) and reaches a plateau in adulthood between 10 and 11 Hz (group 4: *⌀* 22.36 yrs). Consequently, we observe a significant age group effect with regard to the DRF on every selected cerebral region between the adult group and the children samples (*F*(2,28) = 13.287; *P* < .001). However, the various children samples do not differ significantly from each other.

## 4. Discussion

Our results demonstrate pronounced driving responses in all subjects involved in the present study. Since the consulted parameter “accuracy rates” depicts the ratio between correct and incorrect commands, it constitutes an indirect measure of evoked neuronal activity. However, though we do not observe physiologic activity in detail, the course of SSVEP classifications enables to infer the underlying resonance dynamics from the behavioural performance. In the present investigation a fictional longitudinal study of four groups ranging from 7 to about 22 years allows us to reconstruct the functional interplay between development-specific characteristics of the background EEG and varying kinds of evoked SSVEP responses. Thereby we observe differences in the driving profiles apparently in close communication with the specific type of intermittent stimulation. In particular, considerably low classification accuracies within all young samples (7 to 10 years) on the basis of stimulation between 7 and 11 Hz. This supports the assumption that age groups up to (at least) ten years have difficulties to generate phase-locked driving responses coupled to a triggering event in this particular bandwidth; a finding similarly observed in [34] for comparable samples and frequency ranges but on different sensor modalities. The authors emphasize that children up to six years are not yet capable of synchronizing evoked alpha oscillations on adult level. Birca et al. [13] showed that phase alignment serves as a good indicator for SSVEP maturation, especially at occipital areas in children between 7 and 10 years and propose that this phenomenon reflects structural and functional maturation of the involved cerebral regions. These findings may play an important role in the present deficits of younger children to synchronize low frequency steady-state evoked oscillations and thus finds expression in the significant lower classification accuracies. Thereby the deficits are mainly reflected in the range of 10 and 11 Hz. In the adult sample the two of them are accountable for the highest classification rates compared to all other low stimulation frequencies, indicating excellent resonance properties of the underlying neuronal oscillators. As a consequence, the above-average performance of the adult group is determined to a great extent by elaborated driving responses at 10 and 11 Hz, matching exactly the DRF in the spontaneous EEG of the corresponding sample. Accordingly, and as depicted in previous studies in [12, 13] the induced driving seems to be closely tied to the characteristics of the background activity, meaning positively correlated with evolved mechanisms of endogenous frequency synchronization. The younger participants also seem to achieve higher accuracy rates on the basis of 10 and 11 Hz compared to their performance with 7, 8, or 9 Hz stimulation. Though these observations are not yet statistically verified, it suggests that the proposed oscillator rudimentarily exhibits its resonance properties already at an early stage.

It is generally accepted that the occipital alpha rhythm increases in frequency from about 8 to 11 Hz between infancy and adolescence [4, 35]. Nuñez et al. [36] propose that this increase is related to corticocortical myelination during brain maturation. Our present findings also show a developmental increase of the DRF from just over 8 Hz (7 years) to little more than 9 Hz (10 years), finally leveling out between 10 and 11 Hz (adults). It remains unclear whether the same neuronal components are responsible for both, the generation of spontaneous and evoked activity (as proposed by [12]); still, we state a parallel evolution between the ability to synchronize spontaneous and steady-state evoked oscillations in the frequency range of 10 and 11 Hz. Birca et al. [15] add for consideration that a similar developmental course of two cerebral rhythms not necessarily point to common operating principles; however, in the case at hand it may be accepted that evoked oscillations presupposes adequate endogenous synchronization mechanisms. Consequently, the achievements of the younger samples should approximate progressively to the adult performance as soon as the DRF reaches the plateau among 10 and 11 Hz. Hence, the ability to synchronize alpha activity at this very frequency range seems to precede the ability to control a frequency-based BCI system in the form of a predictor of performance.

Moreover, several studies [2, 15] show that intermittent photic stimulation influences spontaneous activity by selectively suppressing the DRF. Besides the limited capacity of the children sample to synchronize evoked activity in the upper alpha range, a not yet fully developed mechanism of suppression may as well contribute to the poor results by increasing the risk of misclassifications through adjacent frequencies. Anyway, this must remain a hypothesis since our data does not allow a verification of this assumption.

It is reasonable to assume that the observed age-group differences may also arise from development-specific deficits in dealing with visual search tasks, as reported (among others) in [37] for children under ten years. Though the ability to proper handling of the spelling field and the corresponding command prompts certainly makes up an influencing variable, the age disparities become apparent as much more as the amount of slow stimulation frequencies increases. This indicates that neuronal oscillators that are subject to considerable age-specific changes are to be found first and foremost in upper parts of the alpha range. As a consequence thereof we do observe only slight group differences upon high frequency stimulation. Although in accordance with parts of the literature available [15] this contradicts the accepted opinion of an increased occurrence of high frequency photic driving with age as an indicator of brain maturation [38]. Yet, in the current investigation we notice that the factor age has a comparatively little effect on the driving responses between 30 and 40 Hz. The comparison of the behavioural performance (accuracy rates) reveals no significant group differences. Recent studies of our team identified a neural oscillator in adults at 32 Hz (unpublished data) with pronounced cortical reactions to flickering stimuli compared to adjacent frequencies. Similar results are reported in [3] with regard to induced driving at 40 Hz. Against the background of the current findings it is indicated that the 32 Hz oscillator is not subject to developmental changes as observed in the lower frequency ranges. However, the present frequency spectrum extends only to 38 Hz; age-specific changes within a 40 Hz oscillator remain unclear.

As to be expected, the amount of aborted attempts (unfinished words) increases as the accuracy level decreases. This is particularly evident in the high drop-out rates of the youngest age group under low frequency stimulation and constitutes a direct reaction to the complete inability to control a BCI adequate to requirements. Therefore it can be emphasized that the factor age gains influence with decreasing stimulation frequency. As a consequence, a child-oriented framework obviously has to go without stimulation close to the alpha range as long as the endogenous synchronization mechanisms prevent induced driving responses on adult level. In contrast, visual stimulation between 13 and 17 Hz (medium frequency condition) in children among eight and nine years already leads to classification accuracies comparable to our findings in grownups. According to [3] frequencies among 15 Hz elicit steady-state oscillations with largest amplitudes. Due to the fact that this frequency range is most effective in eliciting generalized photoparoxysmal responses it can be adopted that visual neuronal networks have a disposition to resonate at this particular frequency [39]. We observe group-specific distinctions in this range only between adults and seven-year-old subjects. This points to age-differentiated mechanisms for neuronal oscillators; however, the resonance properties obviously achieve matured skills much earlier compared to the upper alpha range.

All in all, the medium frequency condition seems to constitute the most suitable SSVEP framework for children of eight years and older with regard to a reliable BCI operation. However, visual annoyance and user fatigue still pose a problem, especially in this specific frequency region. Reports of our youngest subjects subsequent to a session attest this issue (as previously reported in [20] for adult subjects). Therefore and against the background of the observed problems with low and high frequency stimulation, the question of an appropriate SSVEP based BCI system for children remains, similar to the circumstances in adults, purely academic.

## Figures and Tables

**Table 1:** Demographic characteristics of research participants and dominant resting EEG frequency (averaged peak frequency; standard deviation in brackets) on selected locations.

Age group (years)	*n *	Gender ratio (m/f)	Dominant resting EEG frequency (DRF) (⌀ Hz)			
			O_1_	O_2_	PO_3_	PO_4_
Group 1: ⌀ 6.73	11	5/6	8.60 (0.69)	8.60 (1.42)	8.62 (1.24)	8.70 (1.35)
Group 2: ⌀ 8.08	12	3/9	(*x*)	(*x*)	(*x*)	(*x*)
Group 3: ⌀ 9.86	14	11/3	9.24 (0.97)	9.25 (0.99)	9.23 (1.08)	9.27 (0.97)
Group 4: ⌀ 22.36	14	1/13	11.30 (1.03)***	10.88 (0.70)**	10.68 (1.11)**	10.80 (0.96)**

Significance marks: **P < .01 and ***P < .001 denote the levels of significance between group 4 and all other age groups. (*x*) due to a record failure the corresponding data is not evaluable.
